# Selections and Abstracts

**Published:** 1890-01

**Authors:** 


### Selections and Abstracts.

---

In fractured ribs, Dr. Morton, of Philadelphia, recommends a canvas corset in place of the usual adhesive plaster. The corset should extend five inches above and below the fracture with two rows of hooks and eyelets, so that it can be laced tight.—*Times and Register.*

---

The report of the New York Analyst of drugs shows that the chances for getting drugs of good quality on prescription is 43.8 per cent.; fair, 17.4; inferior, 26; NOT AS CALLED FOR, 11.6; excessive strength, 1.2.—*Times and Register*, Philadelphia, December 7, 1889.

